# The Impact of Rocuronium and Sugammadex on Length of Stay in Patients Undergoing Open Spine Surgery: A Propensity Score-Matched Analysis

**DOI:** 10.3390/bioengineering10080959

**Published:** 2023-08-12

**Authors:** En-Bo Wu, Yan-Yi Li, Kuo-Chuan Hung, Amina M. Illias, Yung-Fong Tsai, Ya-Ling Yang, Jo-Chi Chin, Shao-Chun Wu

**Affiliations:** 1 Department of Anesthesiology, Kaohsiung Chang Gung Memorial Hospital, College of Medicine, Chang Gung University, Kaohsiung 833, Taiwan; enbofive@gmail.com (E.-B.W.); yanyi6325@gmail.com (Y.-Y.L.); inr453@adm.cgmh.org.tw (Y.-L.Y.); 2 School of Medicine, College of Medicine, National Sun Yat-sen University, Kaohsiung 804, Taiwan; ed102605@gmail.com; 3 Department of Anesthesiology, Linko Chang Gung Memorial Hospital, Graduate Institute of Clinical Medical Sciences, College of Medicine, Chang Gung University, Taoyuan 333, Taiwan; aminailliasma@gmail.com (A.M.I.); l12084@adm.cgmh.org.tw (Y.-F.T.); 4 Department of Anesthesiology, Park One International Hospital, Kaohsiung 813, Taiwan; jochi731@gmail.com

**Keywords:** bispectral index, enhanced recovery after surgery, length of stay, rocuronium, spine surgery, sugammadex

## Abstract

Enhanced Recovery After Surgery (ERAS), an all-encompassing perioperative care approach, has been demonstrated to enhance surgical results, mitigate postoperative issues, and decrease the length of hospital stay (LOS) in diverse surgical specialties. In this retrospective study, our objective was to examine the influence of muscle relaxant selection on LOS and perioperative results in adult patients undergoing open spine surgery. Specifically, we compared 201 patients who received cisatracurium and neostigmine with 201 patients who received rocuronium and sugammadex, after 1:1 propensity score matching. The utilization of the rocuronium and sugammadex combination in anesthesia for open spinal surgery did not lead to a reduction in the LOS but was associated with a decreased incidence of postoperative chest radiographic abnormalities, including infiltration, consolidation, atelectasis, or pneumonia (*p* = 0.027). In our secondary analysis, multivariate analysis revealed multiple determinants influencing the prolonged LOS (>7 days) during open spine surgery. Bispectral index-guided anesthesia emerged as a protective factor, while variables such as excessive intraoperative blood loss and fluid administration as well as postoperative chest radiographic abnormalities independently contributed to prolonged LOS.

## 1. Introduction

Enhanced recovery after surgery (ERAS) is an all-encompassing strategy for perioperative management, engaging patients, surgeons, and anesthesiologists in the optimization of recovery after anesthesia. The first ERAS guidelines were introduced in 2005, specifically designed for colorectal surgery [1]. Since then, numerous collaborative guidelines and practices have been developed across various surgical disciplines. In 2021, comprehensive guidelines were published by an international group of experts with extensive experience in lumbar spinal fusion, under the guidance of the ERAS Society [2].

Frequently used during spine surgery to facilitate endotracheal intubation and enhance surgical conditions, neuromuscular blocking agents (NMBAs) have been associated with postoperative residual neuromuscular blockade. This condition, however, has been linked to impaired pharyngeal and pulmonary function [3], leading to severe complications such as aspiration, oxygen desaturation, atelectasis [4], and pneumonia [5]. Conventional anticholinesterase-based reversal agents such as neostigmine exhibit limited efficacy in reversing moderate-to-deep or even intense neuromuscular blockade. Sugammadex, as an alternative to neostigmine, efficiently reverses the neuromuscular blockade caused by rocuronium or vecuronium. The use of sugammadex for reversal has demonstrated a reduction in the occurrence of residual paralysis, along with faster reversal, fewer pulmonary complications [6,7], and a lower rate of hospital re-admission [8]. However, upon a thorough review of the current literature, the impact of sugammadex on the LOS for spine surgery remains unknown. This gap in knowledge thus sparked the motivation for our present study.

This investigation was undertaken to fill this void in understanding by retrospectively reviewing the records of patients who had experienced open spine surgery under general anesthesia, using either a combination of cisatracurium and neostigmine or rocuronium and sugammadex. This study also assessed the influence of neuromuscular blockage and reversal drugs on various outcomes, such as intraoperative morphine milligram equivalents (MME), chest radiography abnormalities noted within 7 days after surgery, and the incidence of postoperative nausea and vomiting (PONV).

## 2. Materials and Methods

This retrospective observational cohort study was approved by the institutional review board (IRB) of Kaohsiung Chang Gung Memorial Hospital (IRB approval number: 202101995B0). The trial was documented in line with the Strengthening the Reporting of Observational Studies in Epidemiology statement and adhered to the relevant guidelines [9].

### 2.1. Data Collection and Study Design

**Figure 1:**
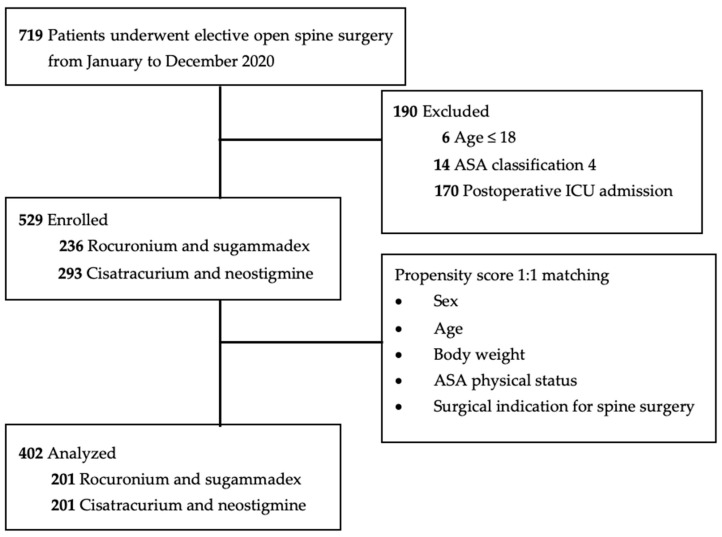
Flow chart of study individuals. ASA—American Society of Anesthesiologists physical status classification; ICU—intensive care unit.

Between January and December 2020, 719 patients underwent elective open spine surgery under general anesthesia at our institution in Southern Taiwan. Under general anesthesia, all patients were given one of two sets of neuromuscular blocking and reversal agents: cisatracurium with neostigmine or rocuronium with sugammadex. As the neuromuscular blocking and reversal agent, cisatracurium and neostigmine were commonly employed. Rocuronium, on the other hand, was given exclusively upon the patient’s consent to an extra cost, around USD 200, for sugammadex. After excluding patients under 18 years of age (*n* = 6), classified as American Society of Anesthesiologists (ASA) physical status 4 (*n* = 14), and admitted to the postoperative intensive care unit (*n* = 170), 529 patients were included in the study. The patients were categorized into two distinct groups: the first was administered rocuronium and sugammadex (*n* = 236) and the second was given cisatracurium and neostigmine (*n* = 293). Ultimately, a 1:1 propensity score-matched analysis, taking into account sex, age, body weight, ASA physical status classification, and indications for spine surgery, was conducted on 402 patients (201 in each group) (Figure 1). Following the application of propensity score matching, the surgical indications for spinal surgery were classified into several procedures: spinal fusion (*n* = 187), decompression (*n* = 45), discectomy (*n* = 120), the removal of spinal cord tumors (*n* = 38), and those among additional categories (*n* = 12).

### 2.2. Anesthesia Management

For patients who underwent open spine surgery, general anesthesia was induced using a combination of intravenous fentanyl (2 mcg/kg), lidocaine (1.5 mg/kg), and propofol (2 mg/kg). Cisatracurium (0.2 mg/kg) or rocuronium (0.8 mg/kg) was administered during induction, respectively, in the cisatracurium-and-neostigmine or rocuronium-and-sugammadex groups, to establish neuromuscular blockade. All patients were intubated and sevoflurane was used to maintain hypnosis during surgery. Regulation of a fresh gas flow of 30–50% oxygen with air was set at 1.5 L/min, adapted based on suitable pulse oximetry values. With the use of the electroencephalographic bispectral index (BIS), the anesthesia depth was monitored and kept within a 40 to 60 range. During the surgical procedure, the objective was to ensure the hemodynamic status remained within a range of ±20% of the initial induction for both groups. A variety of opioids, including fentanyl, alfentanil, and morphine, were selected for use during the procedure, according to the anesthesiologists’ discretion. After the anesthesia was discontinued, the neuromuscular blockade was reversed using sugammadex (2–4 mg/kg) in the rocuronium-and-sugammadex group, or neostigmine (0.05 mg/kg) in the cisatracurium-and-neostigmine group. Since glycopyrrolate was not available at our institution, a combination of neostigmine and atropine (0.02 mg/kg each) was used as a precautionary measure to prevent severe bradycardia or cardiac arrest. Prophylactic measures for PONV were consistently administered according to “Fourth consensus guidelines for the management of postoperative nausea and vomiting [10] during anesthesia induction or before the completion of the surgery, based on individual risk factors.

### 2.3. Primary and Secondary Outcomes

In this study, the time from the end of surgery to hospital discharge was considered as the primary outcome, referred to as LOS. As secondary outcomes, we evaluated and compared three perioperative measures between both groups: intraoperative MME, chest radiography abnormalities noted within 7 days after surgery, and the incidence of PONV. At our institution, intravenous opioids such as fentanyl, alfentanil, and morphine are commonly used during spine surgery. To ensure a consistent comparison, all doses of intravenous opioids were converted to MME [11]. Certified radiologists assessed all postoperative chest radiographs, discerning and documenting any anomalous findings, which encompassed infiltration, consolidation, atelectasis, or pneumonia.

### 2.4. Statistical Analyses

To assess the potential confounding effects of baseline patient characteristics on the outcomes of interest, a 1:1 propensity score matching was performed using a logistic regression model with sex, age, body weight, ASA physical status, and indications for spine surgery as covariates. Following matching, 85% of the patients in the rocuronium-and-sugammadex group were retained, resulting in the allocation of 201 patients from the cisatracurium-and-neostigmine group. Categorical variables such as sex, ASA physical status, Apfel score, comorbidities, and indications for spine surgery were presented as raw numbers or percentages and compared using Fisher’s exact or chi-square tests. Continuous numeric data were presented as the median (interquartile range, IQR, 25–75%) and compared using either the Student’s *t*-test or the Mann–Whitney U test, depending on normality. The Kolmogorov–Smirnov test was used to assess the normality of the distribution. The impact of each variable on prolonged LOS was evaluated using univariate analysis and multiple logistic regression models, specifically binary logistic regression. Statistical analysis was conducted via SPSS^®^ version 22.0 (IBM^®^ Corp., Armonk, NY, USA), with the threshold for statistical significance established at *p* < 0.05.

## 3. Results

Table 1 and Table 2 present a comprehensive summary of the demographic and clinical characteristics of the participants enrolled throughout the various perioperative phases. No significant differences were observed between the two groups concerning sex, age, body weight, ASA physical status, Apfel score, hypertension, diabetes mellitus, and cerebrovascular accident (Table 1). Additionally, surgical indications for spine surgery were categorized into five types: spinal fusion, decompression, discectomy, excision of spinal cord tumor, and others. However, there were no significant differences in the distribution of these five types of surgeries between the two groups.

In terms of intraoperative variables (Table 2), the rocuronium-and-sugammadex group had a significantly greater use of BIS monitoring during anesthesia (*p* < 0.001) and higher urine output than that in the cisatracurium-and-neostigmine group (1.41 mL/kg/h vs. 1.17 mL/kg/h, *p* = 0.037), even though the urine output for both groups went beyond the typical range of 0.5–1.0 mL/kg/h. In terms of postoperative variables, the rocuronium-and-sugammadex group had a significantly shorter LOS than that of the cisatracurium-and-neostigmine group (6.0 [4.0–8.0] days vs. 7.0 [5.0–10.0] days, *p* < 0.001; Table 2). The combination of rocuronium and sugammadex was associated with a reduced rate of postoperative chest radiographic abnormalities (*p* = 0.027). Additionally, no significant differences were noted between the two groups regarding the duration of anesthesia, blood loss, intraoperative sevoflurane consumption, MME, fluid administration, or the incidence of PONV within either the post-anesthesia care unit (PACU) or the ward.

To perform quantitative analyses, we used both univariate and multiple logistic regression analyses to investigate the independent risk factors associated with prolonged LOS (>7 days, *n* = 402) (Table 3). Univariate analysis revealed that age, ASA physical status 3, duration of anesthesia, sevoflurane consumption, intraoperative fluid administration, blood loss, and postoperative chest radiographic abnormalities were associated with an increased risk of prolonged LOS. In the univariate analysis, the use of a combination of rocuronium and sugammadex, along with intraoperative MME, showed a decreased odds ratio (OR). However, in the multivariate analysis, these were not identified as independent factors associated with a prolonged LOS.

In the multiple logistic regression model, we found that the BIS-guided anesthesia was associated with a lower OR for prolonged LOS (OR = 0.37, 95% CI = 0.18–0.73, *p* = 0.005). Furthermore, ASA physical status 3 and postoperative chest radiographic abnormalities were both associated with a 2.51-fold (95% CI = 1.32–4.78, *p* = 0.005) and 7.66-fold (95% CI = 2.27–25.85, *p* = 0.001) increased risk of prolonged LOS, respectively. Additionally, we observed that a 1 mL/kg/h increase in both intraoperative fluid administration and blood loss was associated with a 1.30-fold (95% CI = 1.02–1.64, *p* = 0.031) and 1.49-fold (95% CI = 1.05–2.12, *p* = 0.028) increased risk of prolonged LOS, respectively. Nevertheless, variables including sex, age, body weight, use of either cisatracurium and neostigmine or rocuronium and sugammadex, duration of anesthesia, sevoflurane consumption, urine output, intraoperative MME, and comorbidities, were not recognized as independent risk factors (Table 3).

## 4. Discussion

This retrospective single-center study aimed to compare the effects of rocuronium and sugammadex with cisatracurium and neostigmine in adult patients who underwent open spine surgery. The findings from our study demonstrated a significant correlation between the application of rocuronium and sugammadex and a reduction in LOS (*p* < 0.001), alongside a decreased rate of postoperative chest radiographic abnormalities such as infiltration, consolidation, atelectasis, or pneumonia (*p* = 0.027). However, in the multivariate analysis, the utilization of the rocuronium and sugammadex combination in anesthesia for open spinal surgery did not result in a reduction in the prolonged LOS.

Numerous prospective randomized clinical trials (RCTs) have reported that the maximum LOS for spine surgery may exceed 7 days, depending on factors such as the surgical site, complexity of the surgical technique, the incidence of perioperative complications, and whether a minimally invasive procedure was employed [13,14,15,16,17,18]. Therefore, for our study, we defined prolonged LOS as a duration exceeding 7 days from the conclusion of surgery until patient discharge.

Initially introduced as “fast-track surgery,” ERAS primarily focused on improving the surgical patient’s experience and care quality, with the main objective of reducing LOS. Visioni et al. conducted a meta-analysis of 10 RCTs, which revealed that the average cost in the ERAS group was significantly reduced by approximately USD 5000 compared with the control group; this was attributed to the reduction in LOS and postoperative readmission rates [19]. According to the ERAS guidelines for consensus statement for perioperative care in lumbar spinal fusion [2], several perioperative factors, including preoperative malnutrition [20], anemia [21], intraoperative hypothermia leading to blood loss [22], delayed postoperative mobilization [23], and PONV [10], have been reported to be associated with LOS. Additionally, Yuk et al. conducted a 13-year retrospective study involving 587 patients who underwent elective cervical spine surgery. They found a significant association between an ASA score > 2 and prolonged LOS [24]. Our study outcomes align with these findings, demonstrating that identified intraoperative blood loss, excessive fluid administration, and an ASA score of 3 as independent factors contributing to a prolonged LOS of >7 days.

Sugammadex, contingent on the dosage, can effectively reverse moderate, deep, and profound blocks engendered by rocuronium [25]. This action assists in preventing residual neuromuscular blockade, thereby mitigating postoperative chest radiographic abnormalities [26,27], in addition to minor and major pulmonary complications [28,29]. According to the literature search to date, the role of sugammadex in the LOS in spine surgery has not yet been extensively studied and concluded. However, sugammadex has established a significant role in ERAS guidelines of colorectal and bariatric surgery [30,31] compared to traditional NMBA reversal agents. Regarding other surgical domains, a retrospective study from 2021 by Song et al. reported that in elective open lobectomy for lung cancer, the use of sugammadex compared to the traditional reversal agent, pyridostigmine, led to a significant decrease in LOS [32]. The authors attributed this result to the use of sugammadex significantly reducing postoperative pulmonary complications which are widely reported as complications after various types of surgeries and have been confirmed to be associated with re-admission [33] and prolonged LOS [34].

The literature has emphasized the importance of early mobilization, including physical therapy [35,36] and prevention of postoperative pulmonary complications [37,38], as essential factors in reducing the LOS for spine surgery. In our study, the combination of rocuronium and sugammadex showed a significantly shorter LOS than that shown by the combination of cisatracurium and neostigmine (6.0 [4.0–8.0] days vs. 7.0 [5.0–10.0] days, *p* < 0.001). Our study likewise observed a reduction in postoperative chest radiographic abnormalities, dropping from 10.9% in the cisatracurium-and-neostigmine group to 5.0% (*p* = 0.027) in the rocuronium-and-sugammadex group. We speculate that these observations might be associated with the use of sugammadex; nonetheless, a definitive causal relationship requires substantiation through additional prospective studies. Furthermore, our secondary analysis also indicated that postoperative chest radiographic abnormalities increase the risk of prolonged LOS by up to 7.66-fold.

BIS-guided anesthesia is now extensively utilized in clinical anesthesiology, providing several advantages. It reduces awareness with recall during general anesthesia in high-risk patients, avoids unnecessary deep anesthesia, shortens the time to extubation, and improves postoperative recovery in the PACU, including a reduction in PONV [39,40].

Generally, the BIS value, indicating appropriate anesthesia depth, typically ranges between 40–60 or 45–65. However, it is susceptible to interference, leading to potential misjudgments. For instance, insufficient NMBAs causing intraoperative involuntary movements can result in increased detection of electromyography signals (30–300 Hz) [41]. Similarly, a shortage of intraoperative analgesics can lead to delta wave (0.5–4 Hz) arousal in the electroencephalography [42]. In our institute, we utilize the BIS monitor software version 3.50 (Medtronic, Minneapolis, MN, USA), which incorporates an electroencephalographic density spectral array (EEG DSA). This approach enables us to avoid misjudgments solely based on the BIS value and allows us to adjust appropriate hypnotics and analgesics during general anesthesia based on the EEG DSA to preserve the power of alpha-waves (8–12 Hz) [42]. In BIS-guided anesthesia, preserving alpha power guided by EEG DSA has been proven to be beneficial. According to a multicenter study published by Hesse et al. in 2019, the absence of alpha spindles in intraoperative electroencephalography is associated with an increased risk of postoperative delirium (POD) in the PACU [43]. Our multivariate analysis found that BIS-guided anesthesia during open spine surgery reduced the risk of prolonged LOS (OR = 0.37, *p* = 0.005). Several systematic reviews and meta-analyses have concluded that employing BIS-guided anesthesia may lead to a reduction in the incidence of postoperative cognitive dysfunction and POD [44,45,46]. Furthermore, a multicenter RCT conducted by Evered et al. involving 515 patients who underwent major surgery demonstrated a significant association between POD and increased LOS [47]. To date, no literature review has explored the relationship between BIS-guided anesthesia and LOS in spine surgery. Although there is no direct evidence of a correlation between BIS-guided anesthesia and LOS, the findings of our retrospective study will encourage further high-quality research to elucidate the relationship between these two factors.

PONV, having been extensively researched and thoroughly investigated, stands as a significant determinant of the LOS following spine surgery [2]. Prophylaxis or treatment of PONV is crucial in any surgical context. PONV not only extends the LOS, but is also associated with increased medical costs due to subsequent comorbidities. Therefore, preoperative risk assessment using Apfel simplified risk score is essential for spine surgery. The major risk factors include being female, being a non-smoker, having a history of PONV or motion sickness, and using opioids for analgesia postoperatively [48]. Prophylactic therapeutic interventions are recommended for patients at high risk of PONV. However, in the latest edition of the PONV 4th consensus guidelines, sugammadex has not been definitively established as an effective prophylaxis for PONV [10]. In our study, there was no statistically significant difference in the incidence of PONV between the two groups, either in the PACU or the ward. We attributed this finding to the implementation of PONV prophylactic measures according to the PONV consensus guidelines [10], which were tailored to each patient’s risk factors during anesthesia.

### Limitations

Despite matching the propensity scores of both the groups, this retrospective study may still be subject to inherent biases owing to its design. The sample size was limited because the data included only Asian patients who underwent open spine surgery at a single center, which may limit the applicability of the results to open spine surgery alone. In this study, we did not differentiate between cervical, thoracic, lumbar, or mixed spine surgeries. Despite numerous meta-analyses of randomized controlled trials consistently showing that cervical spine surgery has the shortest LOS, sometimes as short as less than 3 days [49,50], in contrast, Grasu et al. reported that the longest LOS for oncological spine surgery at their large cancer center could exceed 30 days [51]. Furthermore, both the extent and type of surgery, such as the number of spinal levels involved and whether it’s oncological spine surgery or simply decompression, can potentially impact intraoperative blood loss and fluid management during the surgical procedure. Finally, at our institution, the combination of cisatracurium and neostigmine remains the preferred choice for NMBA and its reversal agent during general anesthesia. This preference is largely due to the fact that these agents are included in Taiwan’s National Health Insurance coverage. Due to cost-related reasons, sugammadex and BIS monitoring are often used in self-paying patients undergoing general anesthesia. Our study compared only two muscle relaxant combinations, and there may be other combinations or strategies that could also be effective in reducing LOS and improving perioperative outcomes. Therefore, further research is warranted to validate our observations and assess the long-term implications of rocuronium and sugammadex administration in patients undergoing spinal surgery. Nevertheless, our study contributes to the growing literature on ERAS guidelines and highlights the potential benefits of using rocuronium and sugammadex to optimize perioperative care in patients undergoing open spine surgery.

## 5. Conclusions

In this single-center retrospective study, the utilization of the rocuronium and sugammadex combination in anesthesia for open spinal surgery did not lead to a reduction in the LOS but was associated with a decreased incidence of postoperative chest radiographic abnormalities. In our secondary analysis, multivariate analysis revealed multiple determinants influencing the prolonged LOS (>7 days) during open spine surgery. BIS-guided anesthesia emerged as a protective factor, while variables such as excessive intraoperative blood loss and fluid administration, and postoperative chest radiographic abnormalities independently contributed to prolonged LOS.

## Figures and Tables

**Table 1:** Demographic and clinical characteristics of the patients.

Variables (Unit)	N (%) or Median (IQR)	Cisatracurium and Neostigmine *n* = 201	Rocuronium and Sugammadex *n* = 201	*p*-Value
Sex				
Female	178 (44.3%)	94 (46.8%)	84 (41.8%)	0.315
Male	224 (55.7%)	107 (53.2%)	117 (58.2%)	
Age (years)	63 (52–70.3)	62 (51–70.5)	64 (54.5–70.5)	0.449
Body weight (kg)	65 (58–75)	67 (57.5–76)	65 (58–73)	0.230
ASA				
1	2 (0.5%)	1 (0.5%)	1 (0.5%)	1.000
2	184 (45.8%)	92 (45.8%)	92 (45.8%)	
3	216 (53.7%)	108 (53.7%)	108 (53.7%)	
ASA				
1 and 2	183 (46.3%)	93 (46.3%)	93 (46.3%)	1.000
3	216 (53.7%)	108 (53.7%)	108 (53.7%)	
Apfel score				
0	53 (20.1%)	22 (19.5%)	31 (20.5%)	0.430
1	97 (36.7%)	36 (31.9%)	61 (40.4%)	
2	88 (33.3%)	43 (38.1%)	45 (29.8%)	
≧3	26 (9.8%)	12 (10.6%)	14 (9.3%)	
Hypertension				
No	194 (48.3%)	102 (50.7%)	92 (45.8%)	0.318
Yes	208 (51.7%)	99 (49.3%)	109 (54.2%)	
Diabetes mellitus				
No	295 (73.4%)	155 (77.1%)	140 (69.7%)	0.090
Yes	107 (26.6%)	46 (22.9%)	61 (30.3%)	
Cerebrovascular accident				
No	389 (96.8%)	196 (97.5%)	193 (96.0%)	0.398
Yes	13 (3.2%)	5 (2.5%)	8 (4.0%)	
Surgical indications for spine surgery				
Spinal fusion	187 (46.5%)	104 (51.7%)	83 (41.3%)	0.248
Decompression	45 (11.2%)	18 (9.0%)	27 (13.4%)	
Discectomy	120 (29.9%)	57 (28.4%)	63 (31.3%)	
Excision of spinal cord tumor	38 (9.5%)	16 (8.0%)	22 (10.9%)	
Others	12 (3.0%)	6 (3.0%)	6 (3.0%)	

Non-normally distributed data are presented as medians (IQR, 25–75%). Statistical tests such as the Kolmogorov−Smirnov (for normal distribution), Mann-Whitney U, chi-square, and Fisher’s exact tests are used as appropriate. IQR, interquartile range; ASA, American Society of Anesthesiologists physical status classification.

**Table 2:** Clinical manifestations during intraoperative and postoperative periods.

Variables (Unit)	N (%) or Median (IQR)	Cisatracurium and Neostigmine *n* = 201	Rocuronium and Sugammadex *n* = 201	*p*-Value
BIS-guided anesthesia				
No	190 (47.3%)	148 (73.6%)	42 (20.9%)	<0.001
Yes	212 (52.7%)	53 (26.4%)	159 (79.1%)	
Duration of anesthesia (h)	4.58 (3.48–5.98)	4.67 (3.39–6.09)	4.50 (3.49–5.90)	0.647
Sevoflurane consumption (mL/kg/h)	0.17 (0.14–0.21)	0.16 (0.13–0.21)	0.18 (0.16–0.21)	0.059
Sevoflurane consumption (mL)	50.01 (35.63–70.00)	50.01 (35.00–70.14)	50.01 (39.74–69.99)	0.696
Fluid administration (mL/kg/h)	3.99 (3.01–4.92)	4.05 (2.99–4.94)	3.95 (3.03–4.94)	0.985
Urine output (mL/kg/h)	1.28 (0.92–1.79)	1.17 (0.91–1.66)	1.41 (0.92–1.95)	0.037
Blood loss (mL/kg/h)	0.67 (0.30–1.31)	0.82 (0.32–1.34)	0.66 (0.24–1.22)	0.364
Intraoperative MME (mg/h)	3.48 (2.42–4.87)	3.54 (2.46–5.01)	3.47 (2.31–4.63)	0.430
LOS (day)	7.0 (5.0–9.0)	7.0 (5.0–10.0)	6.0 (4.0–8.0)	<0.001
Postoperative chest radiographs				
Normal	370 (92.0%)	179 (89.1%)	191 (95.0%)	0.027
Abnormal ^†^	32 (8.0%)	22 (10.9%)	10 (5.0%)	
PONV at PACU				
No	398 (99.0%)	199 (99.0%)	199 (99.0%)	1.000
Yes	4 (1.0%)	2 (1.0%)	2 (1.0%)	
PONV in the ward				
No	384 (95.5%)	193 (96.0%)	191 (95.0%)	0.630
Yes	18 (4.5%)	8 (4.0%)	10 (5.0%)	

Non-normally distributed data are presented as medians (IQR, 25–75%). Statistical tests such as the Kolmogorov–Smirnov (for normal distribution), Mann–Whitney U, chi-square, and Fisher’s exact tests are used as appropriate. IQR—interquartile range; BIS—bispectral index; MME—morphine milligram equivalent; LOS—length of stay; PONV—postoperative nausea and vomiting; PACU—post-anesthesia care unit. ^†^ Postoperative chest radiographic abnormalities are defined as infiltration, consolidation, atelectasis, or pneumonia.

**Table 3:** Univariate and multivariate logistic regression analysis assessing risk for prolonged LOS (>7 days) (*n* = 402).

Variables (Unit)	N (%) or Median (IQR)	Univariate		Multivariate	
		OR (95% CI)	*p*-Value	OR (95% CI)	*p*-Value
Female	178 (44.3%)	1		1	
Male	224 (55.7%)	0.70 (0.46–1.06)	0.092	0.77 (0.41–1.45)	0.422
Age (y)	63.0 (52.0–70.3)	1.02 (1.00–1.03)	0.031	1.00 (0.97–1.03)	0.847
Body weight (kg)	65.0 (58.0–75.0)	1.00 (0.98–1.01)	0.536	1.01(0.98–1.04)	0.456
BIS					
None	190 (47.3%)	1		1	
Used	212 (52.7%)	0.39 (0.26–0.59)	<0.001	0.37 (0.18–0.73)	0.005
ASA 1 and 2	186 (46.3%)	1		1	
ASA 3	216 (53.7%)	3.24 (2.10–5.01)	<0.001	2.51 (1.32–4.78)	0.005
Cisatracurium and neostigmine	201 (50.0%)	1		1	
Rocuronium and sugammadex	201 (50.0%)	0.43 (0.28–0.65)	<0.001	1.36 (0.68–2.74)	0.388
Duration of anesthesia (h)	4.58 (3.48–5.98)	1.36 (1.22–1.53)	<0.001	1.27 (1.00–1.63)	0.053
Sevoflurane consumption (mL)	50.01 (35.63–70.0)	1.02 (1.01–1.02)	<0.001	1.00 (0.99–1.02)	0.834
Fluid administration (mL/kg/h)	3.99 (3.01–4.92)	1.17 (1.03–1.32)	0.012	1.30 (1.02–1.64)	0.031
Urine output (mL/kg/h)	1.28 (0.92–1.79)	1.12 (0.92–1.36)	0.267	1.26 (0.84–1.90)	0.267
Blood loss (mL/kg/h)	0.69 (0.30–1.31)	1.70 (1.28–2.27)	<0.001	1.49 (1.05–2.12)	0.028
^††^ Intraoperative MME (mg)	3.48 (2.42–4.87)	0.85 (0.75–0.95)	0.004	0.91 (0.74–1.11)	0.362
Diabetes mellitus					
None	295 (73.4%)	1		1	
Yes	107 (26.6%)	1.53 (0.97–2.40)	0.066	0.89 (0.47–1.68)	0.716
Hypertension					
None	194 (48.3%)	1		1	
Yes	208 (51.7%)	1.41 (0.94–2.12)	0.100	1.12 (0.56–2.24)	0.754
^†^ Postoperative chest radiographs					
Normal	370 (92.0%)	1		1	
Abnormal ^†^	32 (8.0%)	11.25 (4.23–29.94)	<0.001	7.66 (2.27–25.85)	0.001

LOS—length of stay; OR—odds ratio; CI—confidence interval; BIS—bispectral index; ASA—American Society of Anesthesiologists physical status classification; MME—morphine milligram equivalents. ^†^ Postoperative chest radiographic abnormalities are defined as infiltration, consolidation, atelectasis, or pneumonia. ^††^ To standardize opioid consumption across different drugs and formulations, opioid consumption is converted into morphine milligram equivalents (MME). The *p*-value is adjusted by controlling the false discovery rate [12].

## Data Availability

The data presented in this study are available from the corresponding author upon request.
